# Electronic and paper versions of a faces pain intensity scale: concordance and preference in hospitalized children

**DOI:** 10.1186/1471-2431-11-87

**Published:** 2011-10-12

**Authors:** Chantal Wood, Carl L von Baeyer, Sylvain Falinower, Dominique Moyse, Daniel Annequin, Valérie Legout

**Affiliations:** 1 Pain Unit, Centre Hospitalier Universitaire Robert Debré, 75019 Paris, France; 2 Departments of Psychology and Pediatrics, University of Saskatchewan, Saskatoon, Canada; 3 A4 SAS Agency, Medical Knowledge Management, 92320 Chatillon, France; 4 Statistician, Paris, France; 5 Pediatric Pain Unit, Armand Trousseau Children's Hospital, 75019, Paris, France; 6 Laboratoires Grünenthal, 92300 Levallois Perret, France

## Abstract

**Background:**

Assessment of pain in children is an important aspect of pain management and can be performed by observational methods or by self-assessment. The Faces Pain Scale-Revised (FPS-R) is a self-report tool which has strong positive correlations with other well established self-report pain intensity measures. It has been recommended for measuring pain intensity in school-aged children (4 years and older). The objective of this study is to compare the concordance and the preference for two versions, electronic and paper, of the FPS-R, and to determine whether an electronic version of the FPS-R can be used by children aged 4 and older.

**Methods:**

The study is an observational, multicenter, randomized, cross-over, controlled, open trial. Medical and surgical patients in two pediatric hospitals (N = 202, age 4-12 years, mean age 8.3 years, 58% male) provided self-reports of their present pain using the FPS-R on a personal digital assistant (PDA) and on a paper version. Paper and electronic versions of the FPS-R were administered by a nurse in a randomized order: half the patients were given the PDA version first and the other half the paper version first. The time between the administrations was planned to be less than 30 minutes but not simultaneous. Two hundred and thirty-seven patients were enrolled; 35 were excluded from analysis because of misunderstanding of instructions or abnormal time between the two assessments.

**Results:**

Final population for analysis comprised 202 children. The overall weighted Kappa was 0.846 (95%CI: 0.795; 0.896) and the Spearman correlation between scores on the two versions was r_s _= 0.911 (p < 0.0001). The mean difference of pain scores was less than 0.1 out of 10, which was neither statistically nor clinically significant; 83.2% of children chose the same face on both versions of the FPS-R. Preference was not modified by order, sex, age, hospitalization unit (medical or surgical units), or previous analgesics. The PDA was preferred by 87.4% of the children who expressed a preference.

**Conclusion:**

The electronic version of the FPS-R can be recommended for use with children aged 4 to 12, either in clinical trials or in hospitals to monitor pain intensity.

## Background

The advantages of data collection in health care using hand-held and notebook computers and personal digital assistants (PDA) are now widely recognized and exploited. These advantages include their ability to capture and transmit immediate (momentary) rather than remembered ratings, reducing recall bias; elimination of the common tendency to omit ratings and backfill them later; a built-in timestamp to ensure accurate recording of the time each rating was made; and preference on the part of most patients for electronic compared with paper-and-pencil measures [1-3]. Electronic data collection has been successfully applied in assessment of pain in adults in both clinical and research settings [4-8]

Within the specialized field of pediatric pain assessment, several research groups have independently programmed electronic devices to obtain patients' self-reports of pain intensity [9-12]. Some of these are designed to upload data to a central computer automatically via a cellular wireless link; others require connection via the internet through a computer; and still others have their data extracted when they are returned to the investigator.

To evaluate these electronic pain scales, a starting point is to compare them with their non-electronic versions. The Faces Pain Scale - Revised (FPS-R) [13,14]http://www.painsourcebook.ca is a self-report scale to measure pain intensity in children aged 4 years or older. To use a faces scale, children need not be able to estimate quantities using numbers (as in numerical rating scales) or distances (as in visual analog scales), but must simply be able to match their pain intensity to one of several pictures of a face showing expressions of varying degrees of pain [15,16] The FPS-R has been widely used and has been considered one of the best tools for self-report of pain intensity in children [16-18].

## Objectives

In the present study, hospitalized children rated their own post-surgical or disease-related pain using the original paper-and-pencil FPS-R, as well as an electronic version of the FPS-R. Concordance and preference for the two versions were compared and associations of scores on the respective versions with age and sex were assessed.

Faces scales are well-established for children 5 years and older. There is debate about whether preschool-age children can give meaningful self-reports on pain scales [15,19]. Thus in the present study we made separate comparisons of the concordance of paper and electronic scores among 4-year-olds with those of older children.

## Method

### Trial design

This is an observational, multicenter, randomized, cross over, controlled, open trial. In France, ethical approval for this type of study (observational without modifying clinical practice), is still not legally required. Nevertheless this study was conducted following ethical rules with respect to parental consent, voluntary participation and confidentiality.

### Participants

Participants were recruited from two hospitals in Paris, France. Inclusion criteria were as follows: age 4 to 12 years; hospitalized in surgical or medical wards; presenting a pain condition (post-operative or disease-related pain); no analgesics administered within 30 min before assessment (except constant infusion); willing to participate. Exclusion criteria were as follows: not able to communicate verbally about their pain; too sedated to respond to questions; emergent or unstable acute pain (as patients were required to be stable on an analgesic protocol).

### Measures

The patients were instructed by a pain specialist nurse using standard French language instructions on how to use the Faces Pain Scale - Revised [13] as shown at http://www.painsourcebook.ca. The original scale on paper consists of 6 drawings of faces, arranged in a horizontal row, with a neutral face at the left (scored 0) and the maximum pain face at the right (scored 10).

**Figure 1:**
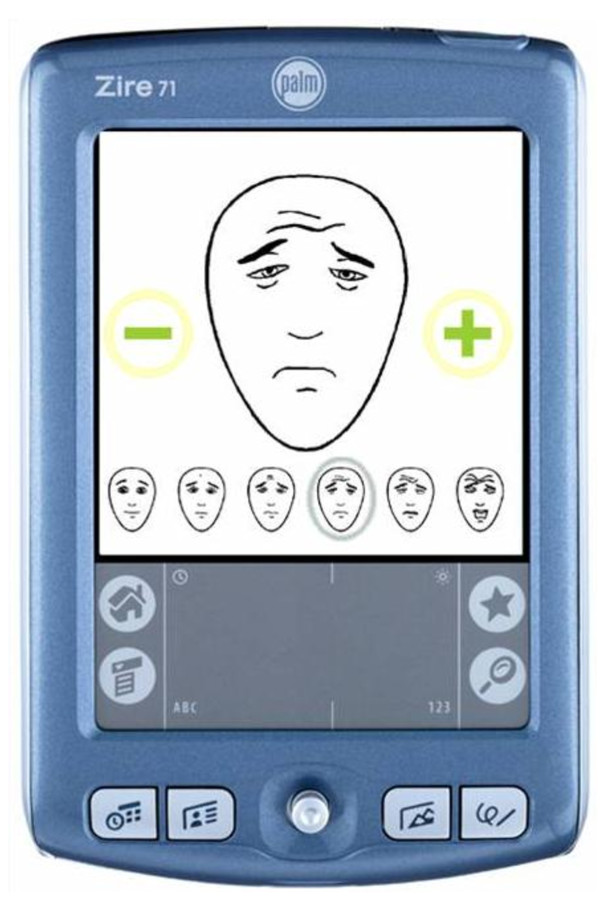
**Electronic version of Faces Pain Scale**. Electronic version of Faces Pain Scale - Revised, using PDA, Palm Zire 71™. Instructions and procedure are in text. The PDA screen measures 55 × 55 mm. The image of the face measures 35 × 25 mm. Faces Pain Scale - Revised: copyright ^©^2001, International Association for the Study of Pain, reproduced with permission. Palm^® ^software: Copyright ^©^2004.

The six faces were reproduced in the same size in a paper version and in an electronic version on a personal digital assistant (PDA), a PalmOne Zire™, as shown in Figure 1.

When the child was first presented with the electronic version, a small version of the six FPS-R faces could be seen at the bottom of the screen but no large face was displayed. When the child pressed (with a finger or stylus) one of the small faces at the bottom, that face was displayed in larger format, together with a plus sign at the right (except for face 10/10) and a minus sign at the left (except for face 0/10). In a 1- to 3-minute period of familiarization with the device, the child was asked to scroll back and forth through the faces by touching the plus and minus symbols on the screen or the small faces in the 6-face display at the bottom. In the present study there was no specific requirement that all of the 6 large versions of the faces be displayed before the actual pain ratings were made, but the full FPS-R scale was visible continuously in the small version, providing a reference to the anchor points. When they said they were ready, participants chose one face corresponding to their current pain intensity. The screen then requested confirmation of the face selected, and a reply (yes or no) had to be given.

### Procedure

Following information and consent of the study, parents were permitted to stay in the room if they wished, but were asked not to discuss the pain ratings with their child. Other persons were asked to leave the room during data collection. The paper and electronic versions of the FPS-R were administered by a nurse in a randomized order: half the patients were given the PDA version first and the other half the paper version first. The time between the administrations was planned to be less than 30 minutes but not simultaneous. The PDA directly recorded the child's response on the electronic version. For the paper version of the FPS-R, after the child indicated a choice of face, the nurse recorded that response. For both versions, the child was asked to point out the face that showed no pain and the face corresponding to maximum pain, in order to ensure that the child could understand the scale; if they did not, the instructions were repeated as needed. The PDA recorded the time for each score. The nurse then asked the child which version she or he preferred, and recorded the preference.

Participants were required to be stable with respect to pain management. In those cases where self-reports indicated severe pain, rescue analgesia or stepped up analgesia was administered as soon as possible while the second assessment was done.

### Outcome

In the present study, hospitalized children rated their own post-surgical or disease-related pain using the original paper-and-pencil FPS-R, as well as an electronic version of the FPS-R. Concordance and preference for the two versions were compared and associations of scores on the respective versions with age and sex were assessed.

### Sample size

A calculation of power and sample size was performed for the difference between paired mean scores on the electronic and paper versions of the FPS-R. Assuming a minimum difference of 1 out of 10 [17] in the mean score, and a standard deviation of 2.5 on each version, with alpha set at 0.05 and power set at 0.90, the number of subjects required would be 68. In order to assess concordance separately for each of the three age groups, that number would be needed for each age group, for a targeted total *N *= 204.

### Randomization

The random allocation sequence was generated by computer on block size of 4.

### Analysis

Statistical analysis was carried out using using SAS version 9.2. Patients' characteristics were tested by non-parametric tests. The difference between PDA and Paper scores was calculated and compared to 0 using a non-parametric test. Agreement between the two methods was assessed by a Kappa statistic weighted by Cicchetti-Allison's procedure. Symmetry for cell proportions used Bowker's test (extending McNemar's test for tables larger than 2 × 2). General characteristics and results on scores were compared for factors likely to impact the results: assessment order, patient's origin (surgery or medicine), sex, and age.

## Results

### Overall population description

The study, including 237 patients, was performed in two pediatric hospitals in France. Thirty-five patients were lost to analysis because of the following reasons: time intervals between the two assessments exactly equal to 0 min. or exceeding 30 min.; misunderstanding of the instructions to use the scale (i.e., inability to point to the lowest and highest levels of pain on the faces scale).

The final population comprised 202 children: 104 started with the electronic version and 98 with the paper version. Patients' characteristics are displayed in Table 1. There were somewhat more males than females (57.9%). The mean age of the patients was 8.3 years (SD = 2.6). The number in each of three age groups was as follows: 4 to 6 years, *n *= 60 (29.7%); 7 to 9 years, *n *= 66 (32.7%); and 10 to 12 years, *n *= 76 (37.6%). Most of the patients came from the surgical department (82.2%). The remainder were medical patients. Eighty percent (80.2%) of the patients were receiving analgesics, without statistical difference between randomized order (p = 0.48) when they were assessed.

**Table 1:** Comparison between randomization groups

	*Palm/Paper (N = 104)*	*Paper/Palm (N = 98)*	*Total (N = 202)*	*p-value*
Sex				
Female	50 (48.1%)	35 (35.7%)	85 (42.1%)	0.088^a^
Male	54 (51.9%)	63 (64.3%)	117 (57.9%)	
Age (years)				
N	104	98	202	
Mean (std)	8.16 (2.50)	8.43 (2.61)	8.29 (2.55)	0.476^b^
Median	8.00	9.00	8.00	
Min; Max	4.0; 12.0	4.0; 12.0	4.0; 12.0	
Age (3 categories)				
4-6	32 (30.8%)	28 (28.6%)	60 (29.7%)	0.922^a^
7-9	33 (31.7%)	33 (33.7%)	66 (32.7%)	
10-12	39 (37.5%)	37 (37.8%)	76 (37.6%)	
Ward where the patient was hospitalized				
Medicine	23 (22.1%)	13 (13.3%)	36 (17.8%)	0.140^a^
Surgery	81 (77.9%)	85 (86.7%)	166 (82.2%)	
Analgesic levels *				
No analgesic	23 (22.1%)	17 (17.3%)	40 (19.8%)	0.015^a^
Level 1	39 (37.5%)	25 (25.5%)	64 (31.7%)	
Level 2	28 (26.9%)	25 (25.5%)	53 (26.2%)	
Level 3	14 (13.5%)	31 (31.6%)	45 (22.3%)	
Analgesic treatment before assessment				
Yes	81 (77.9%)	81 (82.7%)	162 (80.2%)	0.480^a^
No	23 (22.1%)	17 (17.3%)	40 (19.8%)	
Time interval between paper and palm administration (min.)				
N	104	98	202	
Mean (std)	13.57 (6.19)	12.66 (4.74)	13.13 (5.54)	0.258^b^
Median	13.00	12.00	12.00	
Min; Max	1.0; 28.0	4.0; 27.0	1.0; 28.0	
Patient's Preference				
Preference Electronic	85 (89.5%)	74 (85.1%)	159 (87.4%)	0.383^a^
Preference Paper	10 (10.5%)	13 (14.9%)	23 (12.6%)	
'Does not know'	9	11	20	

^a ^: Fisher'exact test

^b ^: Wilcoxon test

* Analgesic levels: reference to the three step analgesic ladder of WHO

The mean time interval between assessments was 13.1 ± 5.5 minutes (range: 1 - 28).

There were no statistical differences in patients' characteristics between the two randomized groups whether they were first assessed by paper or with the PDA device, except for patients treated in the surgery ward who received significantly more level 3 analgesics.

### Pain scores, concordance and preference between paper and electronic versions of FPS-R

The mean levels of the pain scores were 3.1 ± 2.3 and 3.2 ± 2.3 for paper and PDA scores respectively, with no difference related to the assessment order. The mean absolute discrepancy between the two versions was not statistically different significant from zero (0.079 ± 0.953; p = 0.241).

**Figure 2:**
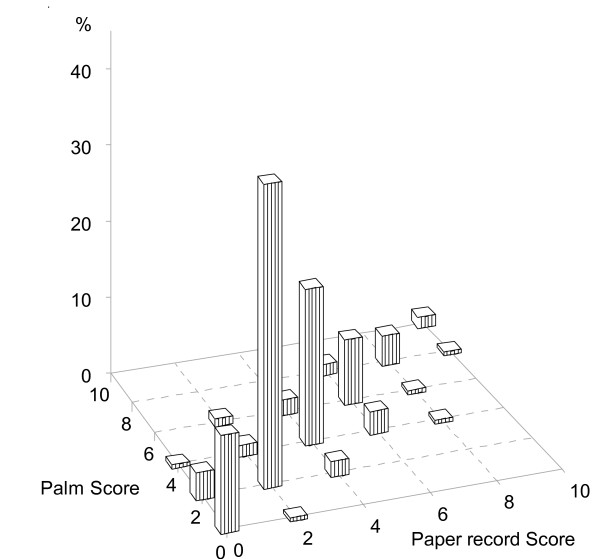
**Distribution of score intensity ratings**. Percentages of score intensity ratings are computed on the overall population (n = 202).

The relationship between scores on the paper and electronic versions of the FPS-R is depicted in Figure 2 in which percentages of all data pairs are represented. In 83.2% of the patients, assessment results were identical whether the assessment was done by paper or PDA device. The measure of agreement (weighted Kappa) reached 0.846 (95% CI: 0.795 - 0.896), which can be considered as very good [20], and the Spearman correlation coefficient on the two versions of the FPS-R was 0.911 (p < 0.0001). The weighted kappa statistics were 0.837 (95% CI: 0.761 - 0.913) and 0.854 (95% CI: 0.786 - 0.921) for electronic/paper and paper/electronic order respectively.

To analyse preference, 20 patients who did not express a preference between electronic versus paper forms were excluded from the analysis for this specific issue. For the other 182 patients, the PDA was preferred over the paper version by 87.4% (Table 1). The preference for the electronic versus paper forms, and the overall difference between electronic and paper assessments did not differ with the order, respectively p = 0.37 and p = 0.52. This trend was observed for all other main characteristics (Table 2).

**Table 2:** Patients' characteristics according to preference

		*Preference Electronic*	*Preference Paper*	*Total*	*p-value^a^*
		(N = 159)	(N = 23)	(N = 182)	
Sex					
	Female	69 (92.0%)	6 (8.0%)	75 (100.0%)	0.173
	Male	90 (84.1%)	17 (15.9%)	107 (100.0%)	
Age (3 categories)					
	4-6 years	45 (81.8%)	10 (18.2%)	55 (100.0%)	0.238
	7-9 years	58 (92.1%)	5 (7.9%)	63 (100.0%)	
	10-12 years	56 (87.5%)	8 (12.5%)	64 (100.0%)	
Ward where the patient was hospitalized					
	Medecine	30 (85.7%)	5 (14.3%)	35 (100.0%)	0.778
	Surgery	129 (87.8%)	18 (12.2%)	147 (100.0%)	
Analgesic levels *					
	No	30 (85.7%)	5 (14.3%)	35 (100.0%)	0.959
	Level 1	49 (86.0%)	8 (14.0%)	57 (100.0%)	
	Level 2	43 (89.6%)	5 (10.4%)	48 (100.0%)	
	Level 3	37 (88.1%)	5 (11.9%)	42 (100.0%)	
Analgesic treatment before assessment					
	Yes	129 (87.8%)	18 (12.2%)	147 (100.0%)	0.778
	No	30 (85.7%)	5 (14.3%)	35 (100.0%)	

^a^Fisher's exact test

* Analgesic levels: reference to the three step analgesic ladder of WHO

The order of the two methods showed no statistically significant differences for the main criteria, they were therefore combined for all subsequent analyses.

### Factors impacting agreement

Agreement between the two methods was calculated by subgroups of the following factors: order of assessment (Electronic/Paper or Paper/Electronic), preference, status, age group, and sex. The results are presented in Table 3. In all cases, weighted kappa statistics were above 0.80. The corresponding mean scores are presented in Table 4: only one statistically significant difference was found between electronic and paper scores in the analysis by sex. In the female subgroup, the electronic mean result was slightly higher than the paper mean result (3.51 vs. 3.27; p = 0.037).

**Table 3:** Weighted Kappa between assessments by factor

*Factor*	*Subgroup*	*Weighted Kappa*	*95% lower limit*	*95%upper limit*
Order	Electronic/Paper (n = 104)	0.837	0.762	0.913
	Paper/electronic (n = 98)	0.854	0.786	0.921
				
Preference^a^	Electronic (n = 159)	0.824	0.761	0.887
	Paper (n = 23)	0.921	0.836	1.000
				
Status	Medicine (n = 36)	0.852	0.745	0.959
	Surgery (n = 166)	0.837	0.777	0.897
				
Age (3 categories)	4-6 years (n = 60)	0.833	0.728	0.939
	7-9 years (n = 66)	0.820	0.725	0.916
	10-12 years (n = 76)	0.869	0.795	0.942
				
Sex	Girls (n = 85)	0.853	0.778	0.928
	Boys (n = 117)	0.838	0.770	0.907
				
Analgesic treatment	None (n = 40)	0.846	0.722	0.970
	Level 1 (n = 64)	0.800	0.705	0.895
	Level 2 (n = 53)	0.835	0.715	0.955
	Level 3 (n = 45)	0.889	0.812	0.966

^a ^Answer 'does not know' was excluded from the calculation of Kappa

* Analgesic levels: reference to the three step analgesic ladder of WHO

**Table 4:** Electronic and paper score by factor

		*Palm score*	*Paper record score*	*Difference^a^*	*p_within_*
Randomization	Palm/Paper (n = 104)	3.13 (2.21)	3.02 (2.25)	0.115 (0.998)	0.315
	Paper/Palm (n = 98)	3.29 (2.30)	3.24 (2.43)	0.041 (0.907)	0.825
	*p_between_*	*0.716*	*0.577*	*0.517*	
					
Preference	Preference Electronic (n = 159)	3.04 (2.11)	2.94 (2.18)	0.101 (1.001)	0.209
	Preference Paper (n = 23)	4.00 (2.95)	4.09 (3.16)	-0.087 (0.733)	1.000
	*p_between_*	*0.159*	*0.136*	*0.375*	
					
Ward	Medicine (n = 36)	4.22 (2.07)	4.33 (2.11)	-0.111 (0.820)	0.688
	Surgery (n = 166)	2.99 (2.23)	2.87 (2.30)	0.120 (0.977)	0.116
	*p_between_*	*< 0.001*	*< 0.001*	*0.169*	
					
Age (3 categories)	4-6 years (n = 60)	2.63 (2.10)	2.53 (2.24)	0.100 (0.933)	0.590
	7-9 years (n = 66)	3.33 (2.39)	3.06 (2.36)	0.273 (1.046)	0.057
	10-12 years (n = 76)	3.55 (2.18)	3.66 (2.30)	-0.105 (0.858)	0.432
	*p_between_*	*0.035^b^*	*0.009^b^*	*0.099*	
Sex	Female (n = 85)	3.51 (2.41)	3.27 (2.37)	0.235 (0.947)	0.037
	Male (n = 117)	2.99 (2.11)	3.03 (2.31)	-0.034 (0.946)	0.811
	*p_between_*	*0.168*	*0.463*	*0.056*	
					
Analgesic treatment	None (n = 40)	3.03 (2.33)	3.00 (2.48)	0.300 (1.067)	0.156
	Level 1 (n = 64)	2.63 (2.07)	2.44 (2.09)	0.188 (0.924)	0.180
	Level 2 (n = 53)	3.47 (1.93)	3.58 (1.98)	-0.113 (0.993)	0.594
	Level 3 (n = 45)	3.64 (2.64)	3.69 (2.70)	-0.044 (0.796)	1.000
	*p_between_*	*0.079*	*0.001^c^*	*0.120*	

^a^Difference equals electronic assessment minus paper assessment

^b^4-6 years group significantly different from 10-12 years group

^c^Level 1 different from Level 2 and Level 3

p_wtihin _: comparison to 0 (sign rank test)

p_between _: Wilcoxon test

* Analgesic levels: reference to the three step analgesic ladder of WHO

Significant statistical differences in the level of pain scores were observed in some subgroups of patients without difference between the two methods: patients hospitalized in medical wards had higher pain intensities (electronic: 4.22; paper: 4.33) than those in surgical wards (electronic: 2.99; paper: 2.87). Older patients had higher pain intensities than younger patients.

### Responses of 4-year-olds

A post hoc analysis was carried out to assess the ability of 4 year olds to assess their pain with the FPS-R. For the 13 patients of the study aged 4 years, the kappa reflecting agreement between electronic and paper version was 0.861, while for children over 4 years (n = 189) it was 0.844.

The mean score on the PDA was 3.54 for the 4 year olds while for the older children it was 3.19. The mean scores for the paper version were 3.38 versus 3.11 for the younger and the older children, respectively. These differences were not significant.

Among the 4-year-olds in the sample, 77% (10/13) had exact agreement of the paper with the electronic scores, compared with 84% (158/189) for all other ages combined.

## Discussion

The object of this study was to compare an electronic and paper version of the FPS-R as measures of pain intensity in hospitalized children aged 4 to 12 in medical and surgical departments. Validation of the electronic version of the FPS-R as a measure of pain intensity was not carried out.

The Faces Pain Scale - Revised was adapted for administration using a personal digital assistant (PalmOne Zire™). The electronic and paper versions were administered to hospitalized children. In all age groups and in both sexes, scores on the two versions were similar. The mean difference of 0.08 between pain scores on the two instruments fell far below the minimum clinically significant difference of approximately 1 out of 10 reported for children using visual analog scales by Powell et al. [21]. Medical patients, who comprised 17.8% of the sample, rated their pain as more intense than the surgical patients by approximately 1 point out of 10. However, the concordance between the electronic and paper versions and the mean difference were very similar in both the medical and surgical subsamples, suggesting that sample differences in level of pain were not reflected in differences in agreement between the electronic and paper versions. Concordance was high in patients with high as well as low pain scores. The difference in mean scores between medical and surgical patients' pain scores could be due to the medical patients presenting more chronic conditions (e.g. sickle cell disease) and more experience with disease-related and procedural pain than the surgical patients, most of whom were in hospital only for brief corrective surgical procedures.

In all age groups and in both sexes, the electronic version was preferred to the paper version by most patients. Does children's strong preference for the PDA over the paper version translate into better compliance with the PDA? A randomized experiment by Palermo et al. [11] showed that children were significantly more likely to complete diary entries in an electronic than in a paper diary, and the electronic entries were significantly less likely to contain errors and omissions.

Of particular interest is that the concordance between the electronic and paper versions, both in terms of Kappa value and low mean difference, was as strong among 4-year-olds as among all other ages within the sample, suggesting that even the youngest children utilized the two versions as reliably as older children.

The results are consistent with those of other investigators. Specifically, Gulur et al. [22] compared a Computer Face Scale with the Wong-Baker FACES Pain Rating Scale, finding support for the concurrent validity of, and preference for, the computer scale. Falinower et al [9] similarly found concordance between the electronic and paper versions of the FPS-R, and preference for the electronic format.

Thus the present data provide further evidence for the concordance between the two versions and acceptability of an electronic version of the FPS-R based on high Kappa values (> 0.80), and low mean differences (less than 1% of the scale range), and strong preference in comparison with the paper version. Separate studies are needed to establish its usability [12] and feasibility [23] in clinical settings. Usability refers to ease of learning and convenience of use of the instrument. Feasibility refers to compliance, technical reliability, cost, and acceptance by staff and patients in real clinical settings.

A principal limitation of the current study is that data on the underlying clinical conditions of the participants and their previous experience with pain and with pain rating scales were not collected. Such data would establish more clearly the generalizability of the findings, and could help to understand the difference in mean pain scores between medical and surgical patients. A further possible limitation is the restricted range of pain intensity. Most patients (80.2%) were receiving analgesics which could explain the relatively low pain scores (overall mean 3.2/10). However, pain intensity did not affect concordance between the PDA and paper versions of the FPS-R. Implementation of PDA-based clinical data collection is not without some perceived and real disadvantages [24]. PDAs are expensive compared with paper, so their use in collecting pain ratings will probably be limited to clinical settings where the devices have additional applications, for example as part of text messaging systems, electronic health records, electronic prescribing, or electronic order entry. Implementing PDAs for such purposes requires a considerable investment of time and training. Loss and breakage of the PDAs, although no problem in the current study, would be likely to occur to some extent in wider clinical use, and the potential cost of lost hardware and data would have to be estimated and factored into planning for adoption of such systems.

Pending further evidence of usability and feasibility, some practical advantages of the PDA scale can be summarized. Clinicians can carry a PDA in their pocket and it can contain many other resources such as medication dosage charts and other reference information. Individual patient pain trajectories over time can be recorded and displayed on the PDA, allowing clinicians and patients to identify patterns of pain requiring analgesic adjustments. Direct access to recorded pain scores would eliminate the bias to report greater pain in retrospective reports for pain over past time periods, which is an important consideration during clinical consultations.

A promising development which may occur in the near future would be adaptation of the FPS-R and other scales for use on cellular telephones, which are increasingly being designed with PDA features ('smartphones'). This would enable patients who are at home with longer-term pain (post-operative or chronic) to have pain diaries and to transmit the information easily by telephone to the clinic [25,26]. An additional advantage of the use of a cellular phone would be that a separate PDA would not need to be purchased to follow pain scores.

Validation would include showing expected response to pain-producing and pain-relieving events, showing concordance with other self-report and observational pain scales, and showing the expected trajectory of healing over time [27]. These represent possibilities for further research.

## Conclusion

Based on the present results, the electronic version of the FPS-R can be recommended for use in clinical trials of analgesics and other pain-related variables in children from 4 through 12 years of age. The advantages of electronic administration, in terms of technical reliability, reduced recall bias, convenience of data acquisition and processing, and patient preference, are expected to be seen with use and further adaptation of this new technology.

## List of abbreviations used

PDA: personal digital assistant; FPS-R: Faces Pain Scale-Revised; CI: Confidence Interval; WHO: World Health Organization.

## Competing interests

The authors declare that they have no competing interests.

## Authors' contributions

CW had full access to all the data in the study and takes responsibility for the integrity of the data and the accuracy of the data analysis. Study design: CW, SF, CvB. Acquisition of data: CW, DA. Analysis and interpretation of the data: DM, CvB, CW, SF, DA. Manuscript preparation: CvB, CW, VL, DM. All authors read and approved the manuscript.

## Pre-publication history

The pre-publication history for this paper can be accessed here:

http://www.biomedcentral.com/1471-2431/11/87/prepub
